# Multidimensional Minimum Euclidean Distance Approach Using Radar Reflectivities for Oil Slick Thickness Estimation

**DOI:** 10.3390/s22041431

**Published:** 2022-02-13

**Authors:** Bilal Hammoud, Georges Daou, Norbert Wehn

**Affiliations:** 1 Microelectronic Systems Design Research Group, Department of Electrical and Computer Engineering, Technical University of Kaiserslautern, 67663 Kaiserslautern, Germany; wehn@eit.uni-kl.de; 2 Department of Electrical and Computer Engineering, School of Engineering, Lebanese American University, Byblos P.O. Box 36, Lebanon; georges.daou@lau.edu

**Keywords:** oil spill, slick thickness, wide-band radar, power reflection coefficient (reflectivity), constellation sets, multi-frequency estimators, iterative procedure

## Abstract

The need for oil spill monitoring systems has long been of concern in an attempt to contain damage with a rapid response time. When it comes to oil thickness estimation, few reliable methods capable of accurately measuring the thickness of thick oil slick (in mm) on top of the sea surface have been advanced. In this article, we provide accurate estimates of oil slick thicknesses using nadir-looking wide-band radar sensors by incorporating both C- and X-frequency bands operating over calm ocean when the weather conditions are suitable for cleaning operations and the wind speed is very low (<3 m/s). We develop Maximum-Likelihood dual- and multi-frequency statistical signal processing algorithms to estimate the thicknesses of spilled oil. The estimators use Minimum-Euclidean-Distance classification problem, in pre-defined multidimensional constellation sets, on radar reflectivity values. Furthermore, to be able to use the algorithms in oil-spill scenarios, we devise and assess the accuracy of a practical iterative procedure to use the proposed 2D and 3D estimators for accurate and reliable thickness estimations in oil-spill scenarios under noisy conditions. Results on simulated and in-lab experimental data show that M-Scan 4D estimators outperform lower-order estimators even when the iterative procedure is applied. This work is a proof that using radar measurements taken from nadir-looking systems, thick oil slick thicknesses up to 10 mm can be accurately estimated. To the best of our knowledge, the radar active sensor has not yet been used to estimate the oil slick thickness.

## 1. Introduction

Oil spills are regrettably common, and have socio-economic implications on communities and disastrous consequences on the marine ecosystem and maritime life [1,2]. They occur due to intentional petroleum waste spill or due to involuntary reasons such as ship accidents and underwater pipeline ruptures [3]. Once a spill occurs, the oil will spread quickly on the water surface to form a layer, know as an “oil slick”. To alleviate the severity of oil spills and promptly react to such incidents, it is crucial to have oil-spill monitoring systems which enable an effective contingency plan [4]. This plan should dictate the best actions to deal with oil spills. Therefore, monitoring systems must perform several functionalities and provide valuable information to contain the damage [5] with a rapid response time allowing for a quick and efficient intervention:

- Oil-spill detection: this provides information about the location of oil slicks, and how large the spread area is. It is necessary information to allow oil spill mapping for both tactical and strategic countermeasures.
- Oil-thickness estimation: the thickness distribution of spilled oil is critical information for spill-containment because it allows an estimation of the total volume spilled, so that adequate tools can be used in clean-up operations.
- Oil-classification: knowledge about oil type is helpful to estimate the environmental damage and appropriate response.

Among the three key pieces of required information, oil type is the least critical one at early stages of the spill. With respect to spill detection, multiple techniques using various technologies and sensors were developed, with the most recent being based on satellite and airborne remote sensing [6,7]. However, when it comes to oil thickness estimation, although both countermeasures effectiveness assessment and legal purposes for prosecution necessitate information of slick thickness. Only few reliable methods capable of accurately measuring the thickness of the oil on top of the sea surface have been advanced [8]. This makes the measurements of slick thickness problematic.

Oil spills can have varying thicknesses, ranging from less than a micrometer (μm) for sheens to millimeters (mm) for thick slicks as documented in the Deep Water Horizon case field samples [9,10]. The techniques available and thoroughly researched almost all tend to measure oil sheens (0.01 to 10μm thickness) [11], although this range does not relate to most oil spill needs, and it is not considered as damaging as thick oil slicks. For effective oil spill clean-up operations, it is crucial to get the thickness measurements greater than 0.5 mm up to about 10 mm [12]. Since more than 90% of spilled oil could remain circumscribed in a region comprising less than 10% of the spill area [13], this again highlights how critical it is to collect information about the thickness of the slicks where most of spilled oil is concentrated.

The challenges of remotely measuring oil slick thickness are examined in details by Fingas [11]. Visual appearance techniques which correlate oil thickness to appearing color are limited to sheens and rainbow-colored slicks due to multiple constructive and destructive light interferences [8,12]. The brightness of the infrared sensing-based imagery does not vary with slick thicknesses. Therefore, we cannot rely on infrared sensors to yield slick thickness measurements [12,14]. Acoustic traveltime is another available method to remotely measure oil thickness [15]. It is based on the calculation of the Doppler shift witnessed by an originally transmitted laser signal, then reflected back from the oil-water structure. Then, the traveltime is used to relate to the thickness value of the oil layer [12]. Fingas reported the ability of one acoustic signal-based prototype system to measure 6 mm thick slicks [11]. However, laser-acoustic sensors are bulky, expensive, require dedicated aircraft, and cannot work in fog or cloud [7]. Alternatively, the passive microwave radiometry sensor [16,17,18,19] has been used long time ago as an indicator of oil slick thickness. The main issue with this technology tends to be the cyclical relation between the microwave brightness of the slick with its thickness, i.e., one signal brightness could imply several possible thicknesses. This obstacle can be overcome by using multiple frequencies. However, current available models can only measure limited thickness ranges [11]. For example, using the aircraft-borne multi-frequency passive microwave radiometer, the measured thickness range is limited between 0.1 and 1.5 mm [13]. Skou suggested to use microwave radiometer and carried out experiments to measure oil thicknesses based on the brightness temperature [17]. The results were underestimating the real thickness values in some cases. The calibration methodology and the selection of frequencies limited the measured thickness to a maximum of 1 mm. The commercial Optimare three- to five-channel microwave instruments, which are the only tools currently available for measuring slick thickness [12], are able to provide thickness up to 3 mm only. In addition to the limited estimated range, the microwave brightness is influenced by other factors, such that weather, sea conditions, and type of oil. Additionally, the requirement of a dedicated aircraft to mount this sensor, its expensive cost, and its low spatial resolution are considered as additional disadvantages of this sensor. Several other researches have tried to advance some techniques concerning the slick thickness estimation but limited development has occurred [12], further stressing on the importance of additional research [11].

To complete the functionalities for an effective contingency plan, it is crucial to provide the oil slick thickness. Therefore, this new work aims at the thickness estimation of thick oil slicks which persist over long time after the spill. The targeted range of thicknesses is between 1 and 10 mm. This full range is not yet covered by the available techniques in the literature. We propose to use single and dual frequencies to estimate a specific thickness value (3 mm) using radar observations [20]. Then, we suggest best frequency pairs for 2D estimators to each thickness value along with an algorithm to use them in practical scenarios [21]. This article extends the previous work to higher dimensions and proves that the new proposed estimators are much better in terms of accuracy in estimation. The new contributions in this article to the oil spill thickness estimation problem are summarized below.

- Using nadir-looking wide-band radar sensors by joint incorporation of C- and X-frequency bands, maximum-likelihood single (1D), dual- (2D), and multi-frequency (3D and 4D), statistical signal processing algorithms are developed to estimate the thicknesses of spilled oil slicks. The estimators use the Minimum-Euclidean-Distance classification problem on radar reflectivity values in pre-defined multidimensional constellation sets. The active radar sensor has not been used to estimate the oil slick thickness so far.
- An advanced practical procedure to use the proposed 2D and 3D estimators for accurate and reliable thickness estimations in oil-spill scenarios under noisy conditions is devised, and its accuracy is tested.
- The performance of the suggested algorithms is tested using simulated and in-lab experimental data. Results show accurate estimates of oil slick thicknesses from 1 to 10 mm.
- The proposed work is a proof of concept and helps take the oil spill-related research work one step forward towards the development of operational tools for oil-spill intervention.

The remaining of this article is organized as follows: Section 2 presents the methods used in the proposed work. It describes the system model and the radar reflectivity parameter. It also derives the proposed multidimensional algorithms, introduces the constellation diagrams for 1D and 2D estimators, and devises an iterative procedure to apply them. Section 3 shows results obtained on simulated and experimental data and discusses the performance of all estimators. Section 4 concludes the work and presents the future perspective of the presented approach.

## 2. Methods

### 2.1. System Model

Once a spill occurs, the oil will spread quickly on the water surface. Many components of heavy oils, such as crude oil which belongs to 35% of the products spilled, will not evaporate at all even over long periods of time and at high temperatures [22]. Moreover, heavy crude oils will not disperse naturally to any significant extent, and they will spread to slicks as thick as several millimeters [10,23]. To physically model the oil slicks on top of the sea surface, we consider a multi-layer structure (air, oil, and sea water) where each layer has its own electrical properties and physical characteristics. The media are assumed to be nonmagnetic. The oil spilled is considered to be thick or heavy so that its thickness is in the mm range [6]. Such a thick layer at open ocean space with very low wind speed (<3 m/s) dampens capillary and short gravity waves, and calms the smaller waves on sea [23]. Hence, the effect of the oil layer is to smooth the sea surface roughness [24,25] which is a consequence of practical importance for oil-spill scene’s analysis. When the surface is perfectly smooth within the radar cross section, the incident EM wave is reflected along the specular direction which allows to fully collect the reflected power under normal incidence. Therefore, the electromagnetic waves (EM) are assumed to be normally incident on the oil slick surface. At the system-level, the radar operates as a nadir-looking system to allow the full capture of specular component of the reflectivity without losses due to off-nadir back-scattering. The depth of the sea water is assumed to be exceeding 2 m, therefore, we can neglect the radar reflections from the sea-floor. Radar reflection coefficients for the multi-layer structure are calculated in the following section.

#### 2.1.1. Reflection Coefficient Calculation for Multi-Layer Structure

The refractive index *n* for the each medium in the multi-layer structure is $n_{i}=\sqrt{\varepsilon_{i}}$, where $\varepsilon_{1}$, $\varepsilon_{2}$, $\varepsilon_{3}$ are the relative dielectric constants of the air, oil, and sea water, respectively. The reflection coefficients for the different interfaces (air-oil, and oil-water) are calculated using:

(1) $$\rho_{ij}=\frac{n_{i}-n_{j}}{n_{i}+n_{j}}$$

The propagation of EM waves through the second layer (oil) in the multi-layer structure will introduce a phase shift δ which is dependent on the oil physical property (relative dielectric constant $\sqrt{\varepsilon_{2}}$), the frequency of the transmitted electromagnetic wave $f_{k}$, and the thickness of the oil slick $d_{q}$ (we keep the sub-indices *k* and *q* for consistency with the following sections). It is given by:

(2) $$\delta =\frac{2\;\pi }{c}\;f_{k}\;n_{2}\;d_{q}$$

where *c* is the speed of light. The phase shift δ adds exponential terms ($e^{j\delta }$ and $e^{-j\delta }$) to the reflection coefficient of the multi-layer structure, rendering it trigonometric. Across the boundaries of the different layers, where the interaction with EM waves occurs, *E* is conserved. Using the continuity property at these interfaces, the reflectivity (power reflection coefficient) for the three-layer structure [26] is given by:

(3) $$R_{d_{q},f_{k}}={\left|\rho \right|}^{2}=\frac{\rho_{12}^{2}+\rho_{23}^{2}+2\;\rho_{12}\;\rho_{23}\;\cos\left(2\;\delta \right)}{1+\rho_{12}^{2}\;\rho_{23}^{2}+2\;\rho_{12}\;\rho_{23}\;\cos\left(2\;\delta \right)}$$

The reflectivity $R_{d_{q},f_{k}}$ is a trigonometric function with period $T_{R}$ that is dependent on the oil-refractive index and the frequency of the electromagnetic wave. The period is expressed as:

(4) $$T_{R}=\frac{2\;\pi }{\frac{2\;\delta }{d_{q}}}=\frac{\pi \;d_{q}}{\frac{2\;\pi \;f_{k}\;n_{2}\;d_{q}}{c}}=\frac{c}{2\;f_{k}\;\sqrt{\varepsilon_{2}}}$$

The reflectivity shown in Equation (3) actually involves the altered features of the sea surface by the covering oil slick. Basically, it is dependent on the physical properties of the multi-layer structure ($\varepsilon_{i}$), the frequency of the transmitted EM ($f_{k}$), and the thickness of the intermediate layer ($d_{q}$). Since the selection of transmitted EM wave is a parameter that is controlled by the operator on site, and the physical properties of the air and water can be identified, then post-processing the radar reflectivity values will allow to extract the implicit information about the oil layer thickness covering the sea surface. This is more elaborated in the following section.

#### 2.1.2. Relation between the Reflectivity and the Frequency

**Figure 1:**
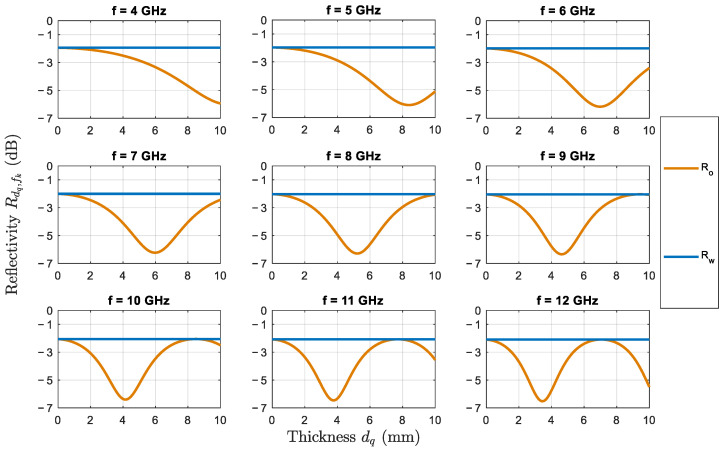
Reflectivity (in dB) versus oil slick thickness (in mm) at different scanning frequencies. $R_{o}$ (= $R_{d_{q},f_{k}}$) and $R_{w}$ are the reflectivity values for oil and water surfaces, respectively.

Since frequencies from the L-band (1–2 GHz) will not change the reflectivity value over the 1–10 mm thickness range by more than 1 dB, they are not considered to be relevant for the proposed approach. Instead, we will use frequencies from the C-band (4–8 GHz) and X-band (8–12 GHz) at increments of 1 GHz for better understanding of the reflectivity behavior at each EM wave frequency. Figure 1 shows the reflectivity value defined in (3) versus the oil thickness which is varied from 1 mm to 10 mm where different subplots correspond to different scanning frequencies. The relative dielectric constant of air is $\varepsilon_{1}=1$. The relative dielectric constant of thick oil is $\varepsilon_{2}=3$, where the imaginary part is of order 0.01 and thus may be neglected [27]. The relative dielectric constant of seawater depends on the water temperature $t_{w}$, the water salinity $s_{w}$ and the frequency of the radar signal. We assume $t_{w}=20$ °C and $s_{w}=35$ ppt [28]. For ease of reference, we also mark the mean reflectivity value in the case of no oil spill as a constant straight line per subplot. For instance, at 4 GHz, the curve of the reflectivity is monotonically decreasing slowly with the thickness. Hence, for small thickness values, the difference between the reflectivity values is very small, so the estimation could go easily wrong. A different pattern is observed at higher frequencies (7–12 GHz). The reflectivity curve admits a steeper slope for small values of thicknesses which improves the estimation of oil slick thickness.

On the other hand, referring to (4), the period $T_{R}$ of the reflectivity is inversely proportional to the frequency of the scanning signal. At large frequencies, $T_{R}$ becomes shorter than the examined range of oil thickness (0–10 mm). Consequently, the same reflectivity value may be attained at different thicknesses which renders them indistinguishable. For instance, the same reflectivity is obtained for 0 mm or 7.2 mm at 12 GHz. The cyclic behavior of the reflectivity might give false estimation at thickness values equal to multiple wavelengths. Therefore, we inspect the need to use the combination of frequency in order to achieve accurate thickness estimation.

### 2.2. Minimum Euclidean Distance Algorithms

The oil slick thickness is denoted by $d_{q}$ mm, where *q* is the index within the plausible discretized range $d_{range}=\left\{d_{min},\ldots ,d_{max}\right\}$. We will use the reflectivity value denoted by $R_{d_{q},f_{k}}$ in Equation (3). It is evaluated when the EM wave’s frequency is $f_{k}$ and when the oil slick thickness is $d_{q}$ mm. To form a pre-defined constellation set for the estimators, and based on the number of frequencies selected, we have to calculate all the reflectivity values for all possible thicknesses in $d_{range}$. Based on the type of the estimator, the constellation set will span a specific number of dimensions. Using single-, dual-, *K*-frequency estimators, the constellation set will span 1, 2, and *K*-dimensions, respectively.

#### 2.2.1. 1D Estimator

Let $\left\{R_{d_{q},f_{k}}\right\}$ be the reflectivity value measured at frequency $f_{k}$ when the oil slick thickness is $d_{q}$. The output of the estimation algorithm is an estimated thickness denoted by $\tilde{d}$. Let $\left\{R_{\tilde{d},f_{k}}\right\}$ be the reflectivity value measured at frequency $f_{k}$ with unknown oil thickness, and leading to an estimated thickness $\tilde{d}$. Therefore, we can define the probability of correctness as:

(5) $$P_{c}=p\left\{\tilde{d}=d_{q}\right\}.$$

The estimator should choose the best index *q* that maximizes $P_{c}$, i.e., it minimizes the difference between $\left\{R_{\tilde{d},f_{k}}\right\}$ and $R_{d_{q},f_{k}}$. Let $o_{d_{q}}$ refer to the event that there is an oil spill on the ocean surface with thickness $d_{q}$, then $p(o_{d_{q}})$ is the probability of the oil slick thickness over $d_{range}$. If the following maximum likelihood rule is satisfied:

$$\begin{array}{c} \left(p\left(\left\{R_{\tilde{d},f_{k}}\right\}|o_{d_{m}}\right)\;.\;p\left(o_{d_{m}}\right)\right)>\left(p\left(\left\{R_{\tilde{d},f_{k}}\right\}|o_{d_{\ell }}\right)\;.\;p\left(o_{d_{\ell }}\right)\right),\;\;\;\forall \left\{m,\ell \right\}\in d_{range},m\neq \ell \end{array}$$

then,

(6) $$\tilde{d}\Rightarrow d_{m}.$$

If all the events of having a thickness $d_{q}$ are equally probable, that is:

$$\begin{array}{c} \;\;\;\;\;\;\;\;\;\;\;\;\;\;\;\;\;\;\;\;\;\;\;\;\;\;\;\;p\left(o_{d_{m}}\right)=p\left(o_{d_{\ell }}\right),\;\;\;\;\;\;\;\forall \left\{m,\ell \right\}\in d_{range},m\neq \ell \end{array}$$

then Equation (6) reduces to:

$$\begin{array}{c} \;\;\;\;\;\;\;p\left(\left\{R_{\tilde{d},f_{k}}\right\}|o_{d_{m}}\right)>p\left(\left\{R_{\tilde{d},f_{k}}\right\}|o_{d_{\ell }}\right),\;\;\;\;\;\;\;\forall \left\{m,\ell \right\}\in d_{range},m\neq \ell \end{array}$$

Defining $P_{e}$, the probability of error in estimation, as:

(7) $$P_{e}=1-P_{c},$$

then, the estimation procedure will turn to a minimization problem. $P_{e}$ is minimized by choosing the thickness in $d_{range}$ which minimizes the minimum Euclidean distance (Δ) between the measured reflectivity $\left\{R_{\tilde{d},f_{k}}\right\}$ and all possible calculated reflectivities $R_{d_{q},f_{k}}$ constituting the constellation set. Hence, $\tilde{d}\Rightarrow d_{m}$ when:

$$\begin{array}{c} \Delta \left(\left\{R_{\tilde{d},f_{k}}\right\},R_{d_{m},f_{k}}\right)<\Delta \left(\left\{R_{\tilde{d},f_{k}}\right\},R_{d_{\ell },f_{k}}\right),\;\;\;\;\;\;\;\forall \left\{m,\ell \right\}\in d_{range},m\neq \ell \end{array}$$

The constellation set for 1D estimator is simply composed of 1D line that represents reflectivity values at each thickness in $d_{range}$ which will be represented by a mapping region. Whenever the noise moves one reflectivity value form one region to another, an error in the estimation will be introduced. As we noticed from the previous discussion, most of the reflectivity plots for the thickness range 1–10 mm have an appearing cyclic behavior. For this reason, it will be a bit hard to elaborate on the constellation set with this cyclic behavior, and we will leave the detailed discussion for higher order estimators. Nevertheless, we can directly tell that due to the periodicity of the reflectivity, there is no unique mapping between relectivities and thicknesses even for an ideal scenario when there is no noise. Hence, unambiguous thickness estimation requires more than a single frequency to be used.

#### 2.2.2. 2D Estimator

When using dual frequencies $f_{i}$ and $f_{j}$, the difference between the measured reflectivity $\left(\left\{R_{\tilde{d},f_{i}}\right\};\left\{R_{\tilde{d},f_{j}}\right\}\right)$ and all other calculated reflectivities $\left(R_{d_{q},f_{i}};R_{d_{q},f_{j}}\right)$ in the constellation set is minimal. Hence, $\tilde{d}\Rightarrow d_{m}$ when:

(8) $$\Delta \left(\left(\left\{R_{\tilde{d},f_{i}}\right\};\left\{R_{\tilde{d},f_{j}}\right\}\right),\left(R_{d_{m},f_{i}};R_{d_{m},f_{j}}\right)\right)<\Delta \left(\left(\left\{R_{\tilde{d},f_{i}}\right\};\left\{R_{\tilde{d},f_{j}}\right\}\right),\left(R_{d_{\ell },f_{i}};R_{d_{\ell },f_{j}}\right)\right).$$

$$\forall \left\{m,\ell \right\}\in d_{range},m\neq \ell$$

2D estimators use 2-dimensional constellation sets composed of different mapping regions “RegX” as estimation regions. Any reflectivity value falling in region “RegX” is mapped to the thickness $\tilde{d}\Rightarrow X\in d_{range}$ mm. From the previous analysis, we noticed that the reflectivity behavior at 4 GHz has a nearly monotonic behavior over $d_{range}$. As the frequency increases, the cyclic behavior of the reflectivity will appear within $d_{range}$. It is clearly observed at 12 GHz when $T_{R}=7.2$ mm. Understanding the cyclic behavior of the reflectivity helps us understanding the shape of the constellation sets for multiple combinations of frequencies.

**Figure 2:**
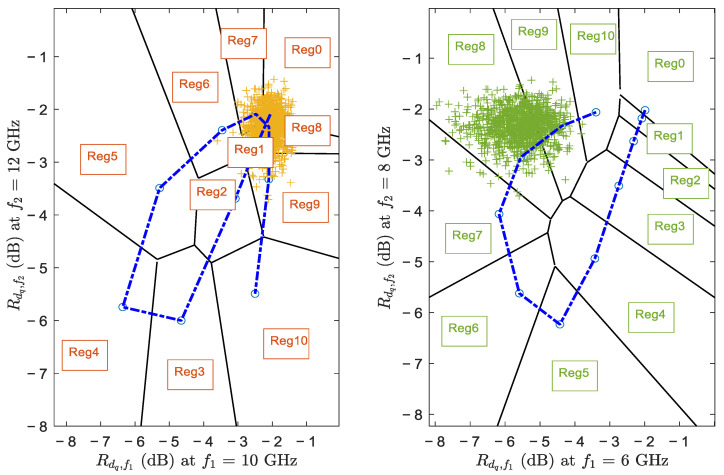
2D Constellation for 8-mm thickness at frequencies $f_{1}$ = 10 GHz and $f_{2}$ = 12 GHz (**left**) and at frequencies $f_{1}$ = 6 GHz and $f_{2}$ = 8 GHz (**right**). Simulated reflectivity values at $d_{q}$ = 8 mm are shown as “+” symbols on the constellations.

In Figure 2, we can see the constellation sets for frequencies ($f_{1}$ = 10 GHz and $f_{2}$ = 12 GHz) (left plot), and ($f_{1}$ = 6 GHz and $f_{2}$ = 8 GHz) (right plot). The dotted line represents reflectivity values for the continuous range of thicknesses from 0 mm to 10 mm. The circle points on this line correspond to discrete values {0,1,…,10} mm. For better understanding, we include 1000 simulated reflectivity values represented by the “+” symbols corresponding to the theoretical thickness $d_{q}$ = 8 mm and under small noise power $\sigma^{2}=0.002$ taken from an additive white Gaussian noise. The majority of reflectivities in the left plot lie within Regs 0–1, and 7–9. This indicates that if we were to estimate an oil with a thickness of 8 mm using the frequency pair {10, 12} GHz, the estimator will sometimes estimate oil thickness as 0 or 1 mm. This is basically due to the small surface area of Reg 8, and to its adjacency to Regs 0 and 1. On the other hand, we can see that in the right plot Reg 8 has a large surface area, is adjacent to Regs 7 and 9, and is away from Reg 0. This is why most estimations will be 8 or 9 mm, reducing the effect of the noise on the estimations. Therefore, now it is clear that the performance of the 2D estimator really depends on the selection of frequency pairs.

However, it is also very important to be aware that the frequency pairs might change with the targeted thickness (which is unknown for us). For example, if the actual oil slick thickness is 1 mm, even though the constellation corresponding to {6,8} GHz was good for a thickness of 8 mm, we might still get estimations of 10 mm because Reg 1 is adjacent to Reg 10 as shown in the right plot of Figure 2. Hence, there should be a best frequency pair for each possible thickness.

This leads to the following two questions:

- Q1. When the oil thickness is unknown, how to select the pair of frequencies?
- Q2. What is the frequency pair that should be selected for the maximum likelihood 2D estimator to estimate a specific oil thickness?

In fact, for each pair of frequencies, we can draw a corresponding constellation set to be used for thickness estimations. Currently we only presented sets without looking at specific thickness values. Q1 is answered in Section 2.3, while Q2 is answered using the iterative procedure in Section 3.2.

#### 2.2.3. K-D Estimator

When using *K* frequencies, the constellation set will be in *K* dimensions, and Equation (8) will be:

(9) $$\begin{array}{c} \Delta \left(\left(\left\{R_{\tilde{d},f_{1}}\right\};\left\{R_{\tilde{d},f_{2}}\right\};\cdots ;\left\{R_{\tilde{d},f_{K}}\right\}\right),\left(R_{d_{m},f_{1}};R_{d_{m},f_{2}};\cdots ;R_{d_{m},f_{K}}\right)\right)\\ <\Delta \left(\left(\left\{R_{\tilde{d},f_{1}}\right\};\left\{R_{\tilde{d},f_{2}}\right\};\cdots ;\left\{R_{\tilde{d},f_{K}}\right\}\right),\left(R_{d_{\ell },f_{1}};R_{d_{\ell },f_{2}};\cdots ;R_{d_{\ell },f_{K}}\right)\right). \end{array}$$

$$\forall \left\{m,\ell \right\}\in d_{range},m\neq \ell$$

#### 2.2.4. Multiple-Scan K-D Estimator

When using *M* observations that are uncorrelated in time, we do the averaging of the measured reflectivity values in the *K*-dimension at each frequency of measurement. Equation (9) becomes:

(10) $$\begin{array}{c} \Delta \left(\frac{1}{M}\sum_{M}\left(\left\{R_{\tilde{d},f_{1}}\right\};\left\{R_{\tilde{d},f_{2}}\right\};\cdots ;\left\{R_{\tilde{d},f_{K}}\right\}\right),\left(R_{d_{m},f_{1}};R_{d_{m},f_{2}};\cdots ;R_{d_{m},f_{K}}\right)\right)\\ <\Delta \left(\frac{1}{M}\sum_{M}\left(\left\{R_{\tilde{d},f_{1}}\right\};\left\{R_{\tilde{d},f_{2}}\right\};\cdots ;\left\{R_{\tilde{d},f_{K}}\right\}\right),\left(R_{d_{\ell },f_{1}};R_{d_{\ell },f_{2}};\cdots ;R_{d_{\ell },f_{K}}\right)\right) \end{array}$$

$$\forall \left\{m,\ell \right\}\in d_{range},m\neq \ell$$

We define the error in the estimation, ϱ, as the absolute value of the difference between the actual thickness *d* and the estimated one $\tilde{d}$:

(11) $$\varrho =|\tilde{d}-d_{q}|.$$

### 2.3. Oil Thickness Estimation Iterative Procedure

In this section, we target to answer Q1 from Section 2.2.2: When the oil thickness is unknown, how to select the pair of frequencies? In the following, we devise a procedure that could be used in real practical scenario. This procedure is iterative, and it relies on developed estimation algorithms so that better and reliable estimations are obtained. To simplify the notations, we will use the 2D estimators in the description of the procedure. Otherwise, it could be easily applied using higher-order estimators as we will see in the analysis later.

#### 2.3.1. Practical Iterative Procedure

We denote by ${\left(f_{1},f_{2}\right)}_{↔d_{q}}$ the best frequency pair to estimate the thickness $d_{q}$ using the maximum-likelihood 2D estimator. We assume that the different frequency pairs are available to the estimation algorithm. Let C be the “collected” estimated thicknesses $\tilde{d}$ whenever considering the delivery of the final estimation. The values in C do not have to be correct estimations. How to assess the accuracy of this procedure is described in the following subsection.

To start the procedure, we use the wide-band radar to scan the area of interest. For each specific area under test, and depending on the radar cross section and the required spatial resolution, the full procedure will run for one time collecting measurements for *M* iterations at *K* frequencies.

Iteration 1: To start the procedure, we define $d_{@0}\in \left\{0:10\right\}$ mm to be a random initial value for the thickness, where (@0) denotes “at iteration #0”. The first estimation is done by the 2D estimator using the pair ${\left(f_{1},f_{2}\right)}_{↔d_{@0}}$ to produce the first estimated thickness at iteration #1: $\tilde{d_{@1}}$. If $\tilde{d_{@1}}=d_{@0}$, then the estimation $\tilde{d_{@1}}$ is added to C, and the algorithm will proceed with the following iterations using the same pair ${\left(f_{1},f_{2}\right)}_{↔d_{@0}}$. Otherwise, $\tilde{d_{@1}}$ is not added to C, and the algorithm will proceed with the following iterations using the pair ${\left(f_{1},f_{2}\right)}_{↔{\tilde{d}}_{@1}}$.

Iteration i: In this iteration, assume that the estimated thickness is ${\tilde{d}}_{i}$. If ${\tilde{d}}_{@i}={\tilde{d}}_{@i-1}$, then the estimation ${\tilde{d}}_{@i}$ is added to C, and the algorithm will proceed with the following iterations using the same pair ${\left(f_{1},f_{2}\right)}_{↔{\tilde{d}}_{@i-1}}$. Otherwise, ${\tilde{d}}_{@i}$ is not added to C, and the algorithm will proceed with the following iterations using the pair ${\left(f_{1},f_{2}\right)}_{↔{\tilde{d}}_{@i}}$.

**Figure 3:**
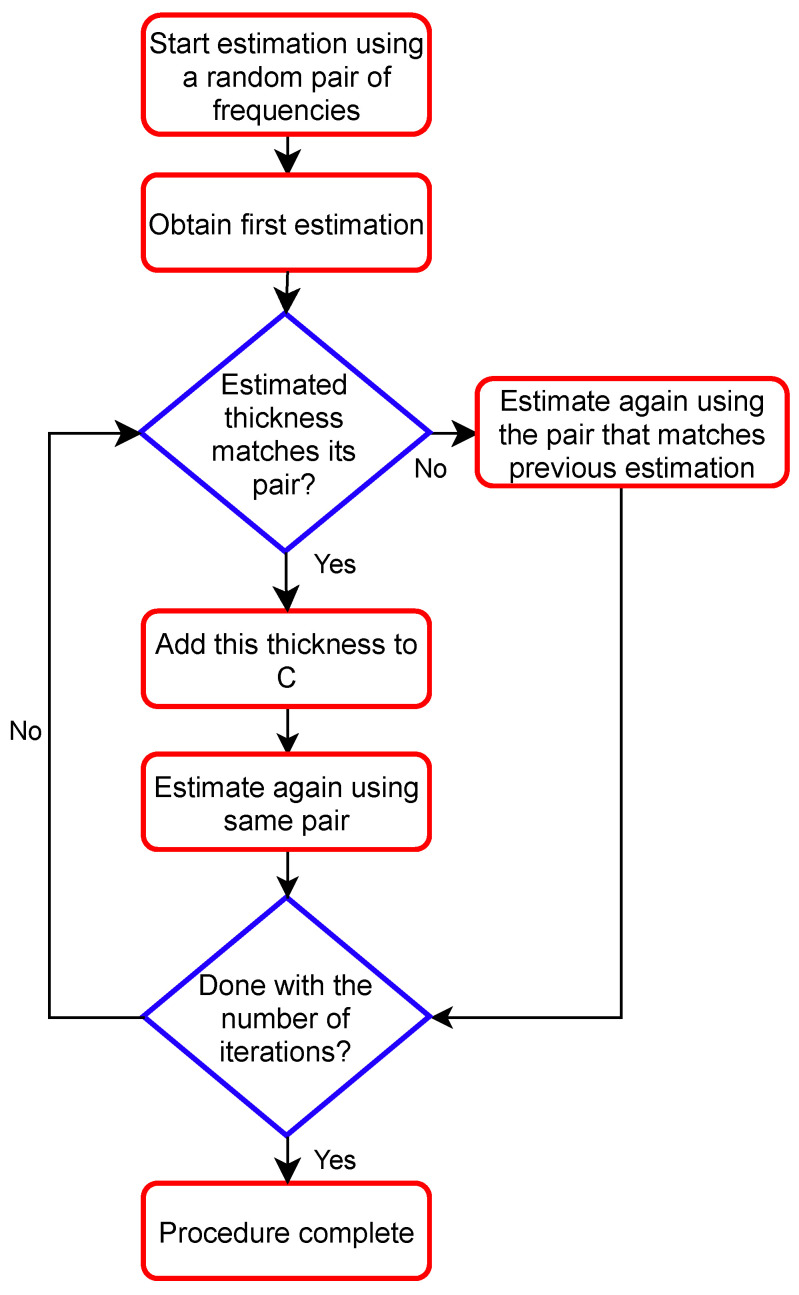
Flowchart of the iterative procedure.

The process is repeated *M* times. We end up with a collection C containing a total number EC of estimated collected thicknesses, where EC is smaller than *M*. The procedure is simplified in the flowchart shown in Figure 3.

#### 2.3.2. Accuracy of the Estimation

To calculate the accuracy of the procedure, we have to count the number of correct estimated thickness collected in the collection C. Define $C_{{\tilde{d}}_{@i}=d_{q}}$ to be the subset of C that includes all the correct estimations: $C_{{\tilde{d}}_{@i}=d}\subseteq C$ where ${\tilde{d}}_{@i}=d_{q}$. The percentage of correctness of this estimator $P\left\{\tilde{d}=d_{q}\right\}$ can be calculated using

(12) $$P\left\{\tilde{d}=d_{q}\right\}=\frac{|C_{{\tilde{d}}_{@i}}|}{|C|}\times 100=\frac{|C_{{\tilde{d}}_{@i}}|}{EC\leq M}\times 100.$$

The algorithm will return ${\tilde{d}}_{m}$ such that $P\left\{{\tilde{d}}_{m}=d_{q}\right\}>P\left\{{\tilde{d}}_{i}=d_{q}\right\}$, ∀${\tilde{d}}_{m}$, ${\tilde{d}}_{i}$∈{0:10}, ${\tilde{d}}_{m}\neq {\tilde{d}}_{i}$ based on Equation (5).

## 3. Results and Discussion

There are too many possibilities in terms of frequency selection and actual thickness under test. In this section, we will focus on one thickness value in order to have a fair comparison between different estimators and to track the improvement in the estimation performance. We will also include the results of 4D estimators for each thickness value. It is important to know that same analysis can be drawn for every possible thickness. For completeness, without loosing the flow in this section, we include detailed results obtained for other thicknesses in the Appendix A.

### 3.1. Simulation Parameters

We evaluate in Matlab the probability of error of the oil slick thickness. The relative dielectric constant of air is $\varepsilon_{1}=1$. The relative dielectric constant of thick oil is $\varepsilon_{2}=3$, where the imaginary part is of order 0.01 and thus may be neglected [27]. The relative dielectric constant of sea water depends on the water temperature $t_{w}$, the water salinity $s_{w}$ and the frequency of the radar signal. We assume $t_{w}=20$ °C and $s_{w}=35$ ppt [28]. The oil thickness is varied from 1 mm to 10 mm. We select scanning frequencies from C- and X-band (4–12 GHz). The thermal noise at the radar equipment is modeled as additive white Gaussian noise of variance $\sigma_{n}^{2}$. We define the following parameters used in the remaining sections: one “simulation” is equivalent to one observation of the oil slick (using one or multiple frequencies at a time depending on the type of estimator). For a single simulation, a corresponding single thickness estimation is calculated by the estimator. The number of “scans” is the number of times the same area is observed by the radar before finally delivering one estimated thickness for the area. The term “iterations” is used in the context of the iterative procedure and has the same meaning of “scans”, except that during the iterative procedure, the estimated thicknesses are generated after each iteration as intermediate values used at the end to get one estimated thickness of the slick. Each “point” in the iterative procedure is an estimation value.

### 3.2. Frequency Pairs and Triads

To deduce the best frequency pairs ${\left(f_{1},f_{2}\right)}_{↔d_{q}}$ corresponding to every thickness $d_{q}$∈{0:10} mm, we use Monte Carlo Simulations in MATLAB. We tested the performance of the 2D estimators using all different frequency combinations $\left(f_{1},f_{2}\right)$, ∀$f_{1}$, $f_{2}$∈ 4–12 GHz (with 1 GHz increment) for every thickness $d_{q}$ separately. The simulations are repeated for different noise powers $\sigma_{1}^{2}=0.01$, $\sigma_{2}^{2}=0.02$, and $\sigma_{3}^{2}=0.002$. Then, the frequency pair corresponding to smallest estimation errors is selected, where we consider an estimation of thickness to be correct if it falls in ±1 mm range of the actual thickness value. The frequency pairs obtained were very similar in all three noise environments, and are listed in Table 1. Note that the frequency pair {6,8} GHz used to plot the right constellation in Figure 2 is indeed the best pair for estimating an actual thickness of 8 mm. We show also in Table 1 the frequency triads that should be used by 3D estimators for each thickness value.

### 3.3. Multiple-Scan 2D Estimators

**Figure 4:**
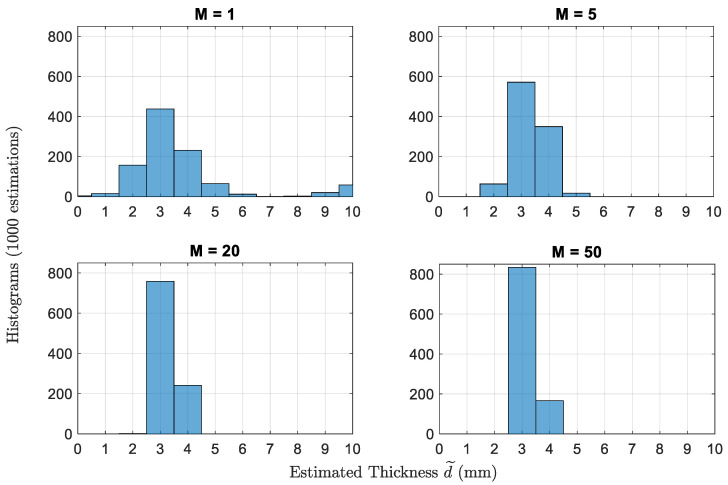
Histograms of the thicknesses estimated by dual-frequency estimator at f=4 GHz and f=12 GHz with multiple scans *M*. The actual thickness *d* is 3 mm.

Figure 4 shows the histograms of the thicknesses estimated by dual-frequency estimator using the best pair $f_{1}=4$ GHz and $f_{2}=12$ GHz with multiple scans *M* when the actual thickness is 3 mm. The estimation is done based on 1000 simulated reflectivity values with noise variance $\sigma_{n}^{2}=0.02$. For single scan (M=1), the probability of error $P_{e}=55\%$, and the maximum error in estimation reaches $\varrho_{max}=7$ mm. $P_{e}$ decreases to 41%,27%, and 18% as *M* increases to 5,20, and 50, respectively. Similarly, $\varrho_{max}$ decreases to 1 mm at M=20 and 50 scans. Obviously, increasing the number of scans over the scene leads to averaging out some AWGN noise samples improving the performance of the estimator. Although the performance is good with 50 scans (82% correct estimations with $\varrho_{max}=1$ mm), but we have to keep in mind that the selection of frequencies was optimized for this specific value of thickness, as if we had some prediction of the actual thickness, which is not realistic in a practical scenario. Therefore, we will use the iterative procedure for the same simulation environment to do the estimations.

**Figure 5:**
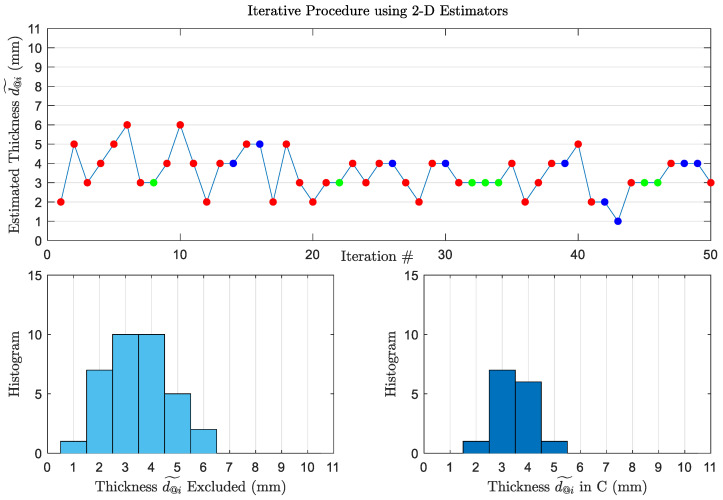
(**Top**): Thickness estimated at each iteration using 50-Scan 2D Estimators. The estimations marked with red are not included in C. The correct estimations marked with green form $C_{{\hat{d}}_{i}=d}$ and are included in C. The estimations marked with blue are included in C, but they are incorrect. The actual thickness is 3 mm. (**Bottom**): Histograms of the estimated thicknesses excluded from C (**left**) and included in C (**right**).

In the upper plot of Figure 5, at iteration #7, the estimated thickness is $\tilde{d_{@7}}=3$ mm. It is obtained by the 2D estimator using the frequency pair corresponding to the estimation ${\left(f_{1},f_{2}\right)}_{↔{\tilde{d}}_{@6}=6mm}$, which is {8,12} GHz ≠{4,12}, which is the best pair for 3 mm ${\left(f_{1},f_{2}\right)}_{↔3mm}$. Therefore, this estimation is marked with red, and will not be included in C. However, at iteration #8, using ${\left(f_{1},f_{2}\right)}_{↔3mm}$, we obtained the estimation 3 mm, so this value is included in C. The histograms of the estimated thicknesses that are added to, or excluded from C are shown at the bottom right and left of Figure 5, respectively. Looking at the histogram of the thicknesses in C (shown at the bottom right of Figure 5), the probability of error $P_{e}$ is 39%. The included wrong estimations are 2,4, and 5 mm, which means that $\varrho_{max}=2$ mm. Looking at the thicknesses excluded from C (shown at the bottom left of Figure 5), many more errors (including the 6-mm estimation) are dropped, which increased the accuracy of the estimator to 61%. If we compare the performance of the procedure compared to the 50-Scan 2D estimator, although the accuracy drops from 82% to 61%, but it is a practical technique which can work during an oil spill for different unknown thicknesses. In addition, although the accuracy is lower, but wrong estimations are far from the actual thickness by 2 mm only. This means that the algorithm helps in rejecting outlier estimations, reducing the effect of noise on the estimator performance.

### 3.4. Multiple-Scan 3D Estimators Using Iterative Procedure

**Figure 6:**
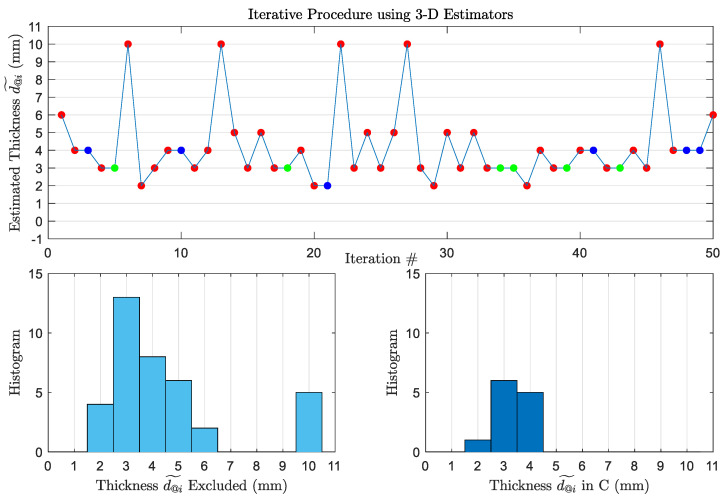
(**Top**): Thickness estimated at each iteration using 50-Scan 3D Estimators. The estimations marked with red are not included in C. The correct estimations marked with green form $C_{{\hat{d}}_{i}=d}$ and are included in C. The estimations marked with blue are included in C, but they are incorrect. The actual thickness is 3 mm. (**Bottom**): Histograms of the estimated thicknesses excluded from C (**left**) and included in C (**right**).

Figure 6 shows the estimated thicknesses at every iteration using 50-Scan 3D Estimators adopting the iterative procedure. Triads are well-known during the procedure, and they are used to get the 3D Maximum-Likelihood estimations. From the top plot, we notice that at iteration #22, the estimation is 10 mm. This estimation is done using the frequency triad ${\left(f_{1},f_{2},f_{3}\right)}_{↔{\tilde{d}}_{@21}=2mm}$ which is {6,12,12} GHz. Looking back at the reflectivity plot at 12 GHz in Figure 1, we see that the 3-mm and the 10-mm thicknesses have the same reflectivity, which explains an insight what could be a possible reason for the selection of 10 mm. However, as shown in the histograms, the procedure is able to reject all 10-mm estimations from entering C because they are outliers. By comparing the performance of 50-Scan 3D estimators to 2D estimators while using the iterative procedure in both cases, we see that $P_{e}$ decreases to from 39% to 24.6%, and $\varrho_{max}$ decreases to 1 mm. Therefore, 3D estimators using the same procedure outperforms the 2D estimators not only by decreasing the probability of error, but also by decreasing the maximum value of the wrong estimations. This estimator is providing estimates with relatively good accuracy, and it is practical for usage in real oil spill scenarios.

### 3.5. Multiple-Scan K-D Estimators

In this section, we evaluate the performance of 4D Minimum-Euclidean-Distance estimators using *M* scans. For a fair comparison to the previous estimators, we will fix the number of scans *M* to 50, meaning that we will repeat the estimation using 4 frequencies 50 times. For the selection of frequencies, since we have now to select 4 values from the range (4–12) GHz, instead of doing the optimization for each thickness value, we will fix the selected frequency values irrespective of the oil thickness. These values are selected more or less uniformly from the range of frequencies, i.e., $f_{1}=4$ GHz, $f_{2}=12$ GHz, $f_{3}=7$ GHz, and $f_{4}=10$ GHz. This way, we get the benefit of the reflectivity characteristics for each of the frequencies in one part of the plausible thickness range (0–10 mm). However, before proceeding with the comparison to previous estimators, let us just first have a look at how would the increase in the number of dimensions alone (without averaging the noise by having multiple scans) affect the performance of estimators.

**Figure 7:**
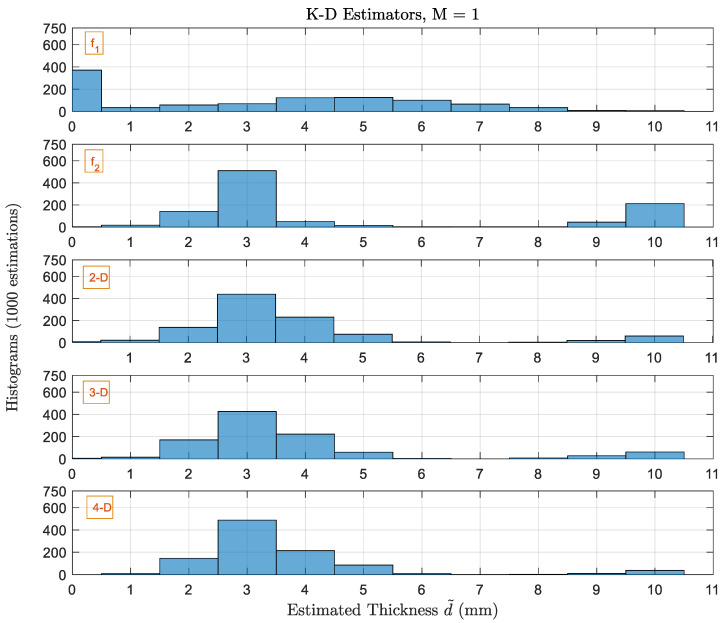
Histograms of the thicknesses estimated by 1D, 2D, 3D and 4D-estimators with single scan. The actual thickness $d_{q}$ is 3 mm.

Figure 7 shows the histograms of 1000 estimated thicknesses by different estimators using single scan (*M* = 1). The actual thickness is again 3 mm. The single-frequency (1D) estimator uses $f_{1}=4$ GHz, or $f_{2}=12$ GHz. The dual-frequency (2D) estimator uses both $f_{1}$ and $f_{2}$ which are the optimal frequency pair for 3 mm as indicated in Table 1. The 3D estimator uses an additional frequency $f_{3}=7$ GHz. The 4D estimator uses $f_{1},f_{2},f_{3}$ in addition to $f_{4}=10$ GHz.

At single scan (*M* = 1), the 1D estimator provides very bad estimations ($P_{e}=92\%$). However, the steep slope of reflectivity at $f_{2}$ decreases $P_{e}$ to 52%, but the ambiguity is there at 10 mm making $\varrho_{max}=7$ mm. $P_{e}$ for the 2D, 3D, and 4D estimators are 55%,58% and 51%, respectively. They do not improve the probability of correctness and they fail to remove the error where $\varrho_{max}=7$ mm. However, higher-order estimators decrease the probability of bigger errors (10 mm) and increases the probability of smaller errors (ϱ=±1 mm). It is worth mentioning here that the 1D estimator is giving good results in terms of the correctness probability compared to higher orders, but the probability of getting $\varrho_{max}=7$ mm is 4 times higher. Yet, we cannot have a conclusive result in terms of better performance for the different estimators.

**Figure 8:**
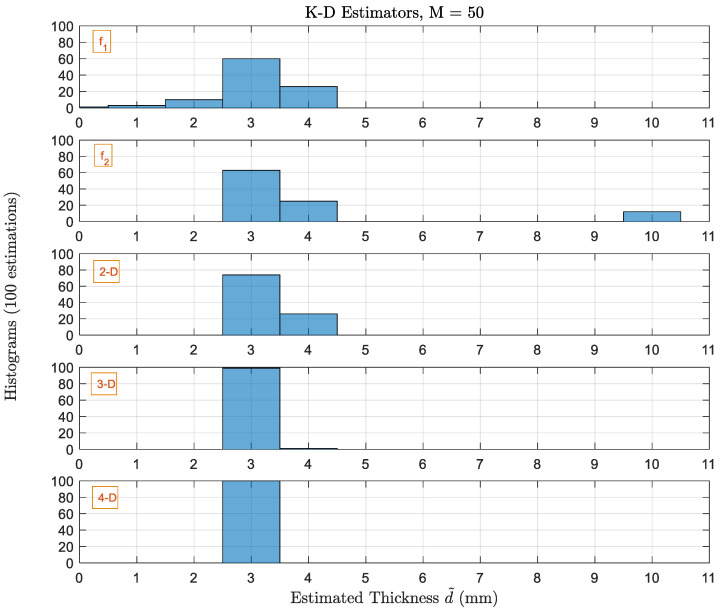
Histograms of the thicknesses estimated by 1D, 2D, 3D and 4D-estimators with M=50 scans. The actual thickness $d_{q}$ is 3 mm.

**Figure 9:**
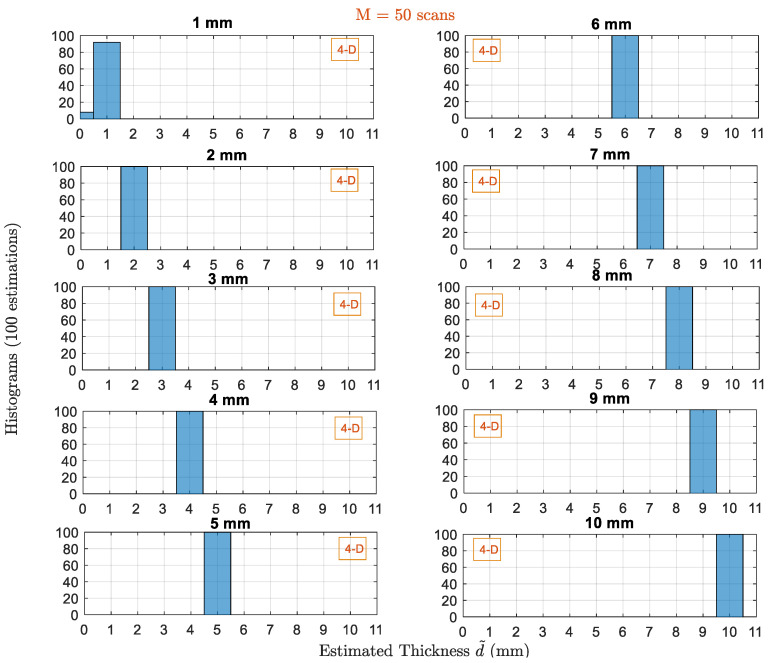
Histograms of the thicknesses estimated by 4D-estimators with M=50 scans for all possible thicknesses $d_{q}$ in $d_{range}$.

Next, we study the performance of same estimators when having multiple scans as shown in Figure 8. When the number of scans increases to 50, the performance ofall higher-order estimators is significantly improved. Whereas we can see that the 1D estimator which provided the less $P_{e}$ with single scan, it is still not able to solve the ambiguity problem. Hence, we still have estimations to 10 mm for 1D at $f_{2}$. $P_{e}$ for the higher-order 2D and 3D estimators decreases to 18% and 1% with $\varrho_{max}=1$ mm. For the 4D estimator, $P_{e}=0\%$. Therefore, 3D and 4D estimators with 50 scans outperform other estimators which use the iterative procedure although the frequencies are not optimized to the specific thickness value. To make sure that the performance of high-order estimator (4D) is not biased to this specific thickness giving extremely good results, we show in Figure 9 the performance of this estimator for all possible thicknesses under the same noise power and using 50 scans. For all thicknesses, the $P_{c}$ is 100% except when the thickness is 1 mm, it is 95%. Therefore, for the 4D estimator with fixed frequency values, with enough number of scans to average out the AWGN noise samples, the effect of the noise is fully canceled, and the estimator is very accurate. More thickness values (1, 5, and 10 mm) are evaluated to make sure that the accuracy of the 4D estimator is only good for specific value. The results are included in the Appendix A to give better insights in different scenarios.

### 3.6. Experimental Results

#### 3.6.1. Experimental Reflectivity Values

Previously, we developed an experimental setup to collect radar reflectivity measurements from an oil-spill lab experiment similar to the one conducted by Daling et al. to get calm-sea conditions for very low wind speeds with no wave action [29]. The experimental setup includes details about the system model with the multi-layer structure, the radar calibration technique, and other setup parameters [30]. During the experiment, a vector network analyzer (VNA) was used to transmit and collect EM waves via directive wide-band Vivaldi antennas (10 cm × 10 cm × 1 mm in length, width, and depth, respectively) pointing normally to the oil surface and operating in the far-field region. The experiment provides reflectivity values at 801 different frequencies from C- and X-bands during a single scan for different oil slicks thicknesses, ranging from 3 mm to 9 mm. Within the context of this work, our goal is to test the accuracy of proposed estimators in Section 2.2 on experimental radar reflectivities. For this, we post-process radar measurements collected from the previous experiment. For consistency, we study the performance of different estimators under the same condition at which results were presented earlier, i.e., when having the actual thickness under test to be 3 mm. A total of 300 measurements (reflectivity values) are collected at each frequency for the 3-mm thickness, i.e., 300 estimations could be obtained. We select 4 frequencies for the 4D estimator to be very close to previously selected frequencies in simulation: $f_{1}=4.01651$ GHz, $f_{2}=11.3119$ GHz, $f_{3}=6.99512$ GHz, and $f_{4}=10.1211$ GHz. We consider a challenging scenario for the performance of the 4D estimator when only one scan is available (M=1).

#### 3.6.2. Experimental Results Analysis

**Figure 10:**
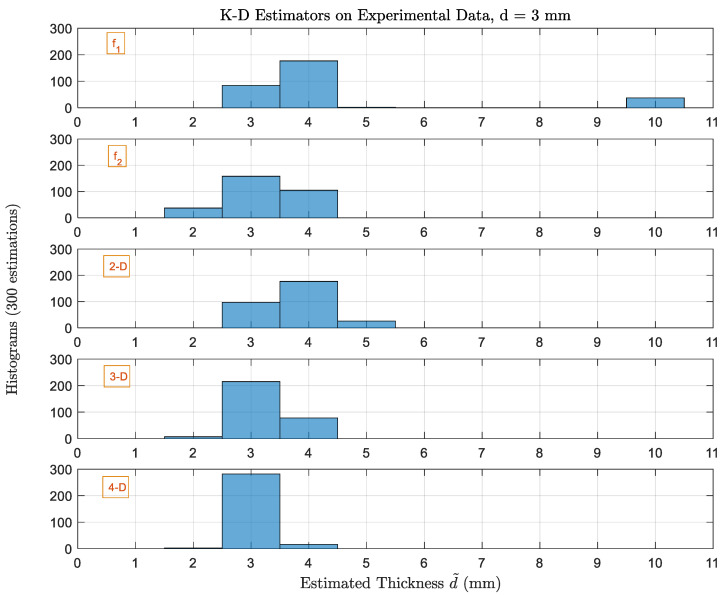
Histograms of estimated thicknesses by 1D, 2D, 3D and 4D-estimators based on experimental reflectivity values with single scan (M=1). The actual thickness $d_{q}$ is 3 mm.

Figure 10 shows histograms of estimated thicknesses by 1D, 2D, 3D and 4D-estimators based on experimental reflectivity values with single scan (M=1). As expected, 1D estimators using single frequency are not providing very good results. Using $f_{1}$, highest estimations are for 4 mm, $P_{e}$ is 72% and $\varrho_{max}=7$ mm. Using $f_{2}$, $\varrho_{max}$ is decreased to 1 mm with $P_{e}$ of 47%. However, single frequency estimators are never used alone, therefore, including $f_{2}$ in the 2D estimator will again increase the $P_{e}$ to 68% but providing the advantage of decreasing $\varrho_{max}$ to 1 mm. For the higher-order 3D estimator, $P_{e}$ decreases to 28% with same $\varrho_{max}$. An observation is that the performance of the 4D estimator for the experimental results looks better than its performance for the simulation data, and this can be explained by the possibility of the high noise power introduced in our simulation environment. For the 4D estimator, the performance is further improved and the $P_{e}$ is reduced to 6%. Thus, it can be said that the higher-order estimators are validated experimentally.

## 4. Conclusions and Future Perspective

Oil-spill monitoring systems must provide valuable information about oil slicks to contain their damage. The estimation of slick thickness is very critical so that adequate tools can be used in the contingency plan. For effective oil spill clean-up operations, it is crucial to get the thickness measurements greater than 0.5 mm up to about 10 mm. Regrettably, available methods are not capable of accurately measuring the thickness of the oil in this full range, which makes the measurements of slick thickness problematic. In this article, we target the estimation of oil slick thickness in this full range. The work provides a new multi-spectral approach using statistical signal processing algorithms to accurately measure oil slick thickness by the use, for the first time, of an active radar sensor. The system model used assumes reflectivity measurements scanned by a wide-band radar sensor operating as a nadir-looking system. We propose dual- and multi-frequency Minimum Euclidean Distance estimators that use known radar power reflection coefficients forming multidimensional constellation sets to estimate the thickness of the oil with single or multiple scans. Increasing the number of reflectivity measurements at different evaluating frequencies increases the percentage of correct estimations. In addition, it decreases the absolute error between estimated and real thicknesses. The performance of the 2D (3D) estimator really depends on the selection of frequency pairs (triads). For each possible thickness, there is be a best frequency pair (triad). Moreover, for the 2D and 3D estimators, we devise a procedure that makes use of the best frequency combinations to increase the accuracy of estimations. It uses optimized pre-defined 2D (3D) constellation sets by utilizing the best pair (triad) of frequencies for each possible thickness value. Then, it processes sequentially the separate estimations done by a Maximum Likelihood frequency estimator in order to optimize the estimation procedure. The performance of the procedure is tested versus higher-order M-scan 4D estimators that select fixed frequencies for scanning. Results show that M-Scan 4D estimators outperform lower-order estimators even when the iterative procedure is applied.

Simulated and in-laboratory experimental results showed high accuracy in estimation. This work is a proof that by processing radar power reflectivity values, taken from nadir-looking systems under weather conditions suitable for cleaning operations, thick oil slick thicknesses up to 10 mm can be accurately estimated. As a future perspective of the presented work, drones would be very convenient platforms for the implementation of the proposed approach. With the proof-of-concept and the performance validation of proposed algorithms on simulated and in-lab experimental data, it is possible to take this work to another implementable level where real drones are used with mounted radar to collect reflectivity measurements for post-processing. Although having nadir-looking radar on drones would produce a smaller radar cross section compared to side-looking systems such as airborne systems, their low cost compared to dedicated aircrafts allows for multiple drones to operate simultaneously. This enables high spatial resolution, quick intervention, wide coverage due to parallelization in scanning, and real-time data collection. Another possible limitation of this approach is its applicability under various meteorological conditions since the developed estimators target calm sea conditions. However, bad weather conditions will not even allow to apply a contingency plan procedure on site. The proposed work is a proof of concept and helps take the oil spill-related research work one step forward towards the development of operational tools for oil-spill intervention.

## Figures and Tables

**Table 1:** 2D (3D) Estimators Best Frequency Pairs (Triads) to Every Thickness.

Thickness	Frequency Pair (2D)	Frequency Triad (3D)
$d_{q}$	${\left(f_{1},f_{2}\right)}_{↔d_{q}}$	${\left(f_{1},f_{2},f_{3}\right)}_{↔d_{q}}$
1 mm	(6 GHz, 12 GHz)	(5 GHz, 12 GHz, 12 GHz)
2 mm	(6 GHz, 12 GHz)	(6 GHz, 12 GHz, 12 GHz)
3 mm	(4 GHz, 12 GHz)	(9 GHz, 9 GHz, 12 GHz)
4 mm	(10 GHz, 11 GHz)	(7 GHz, 9 GHz, 12 GHz)
5 mm	(9 GHz, 9 GHz)	(9 GHz, 12 GHz, 12 GHz)
6 mm	(8 GHz, 12 GHz)	(8 GHz, 10 GHz, 10 GHz)
7 mm	(7 GHz, 10 GHz)	(7 GHz, 9 GHz, 9 GHz)
8 mm	(6 GHz, 8 GHz)	(7 GHz, 8 GHz, 12 GHz)
9 mm	(4 GHz, 8 GHz)	(4 GHz, 12 GHz, 12 GHz)
10 mm	(4 GHz, 12 GHz)	(4 GHz, 11 GHz, 12 GHz)

**Figure A1:**
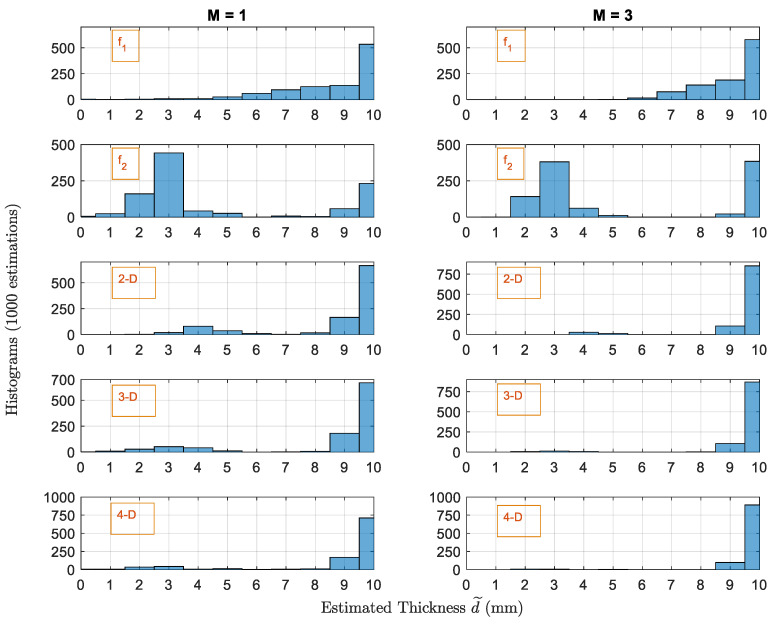
Histograms of the thicknesses estimated by 1D, 2D, 3D and 4D-estimators with single and multiple scans M=3. The actual thickness $d_{q}$ is 10 mm.

**Figure A2:**
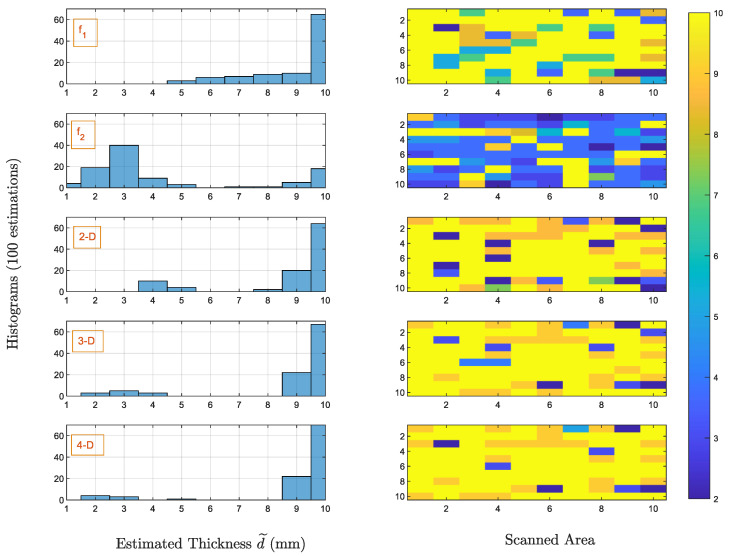
(**Left**) Histograms of the thicknesses estimated by 1D, 2D, 3D and 4D-estimators with single scans M=1. (**Right**) Corresponding maps of scanned area. The actual thickness $d_{q}$ is 10 mm.

**Figure A3:**
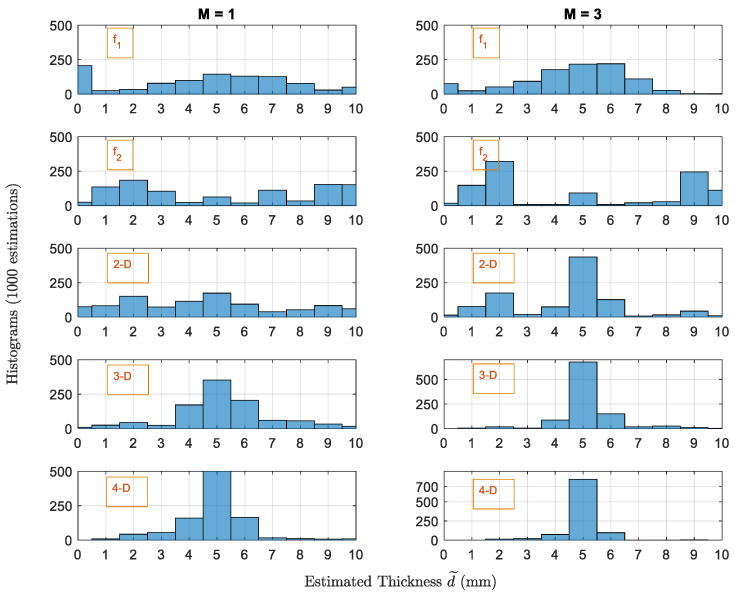
Histograms of the thicknesses estimated by 1D, 2D, 3D and 4D-estimators with single and multiple scans M=3. The actual thickness $d_{q}$ is 5 mm.

**Figure A4:**
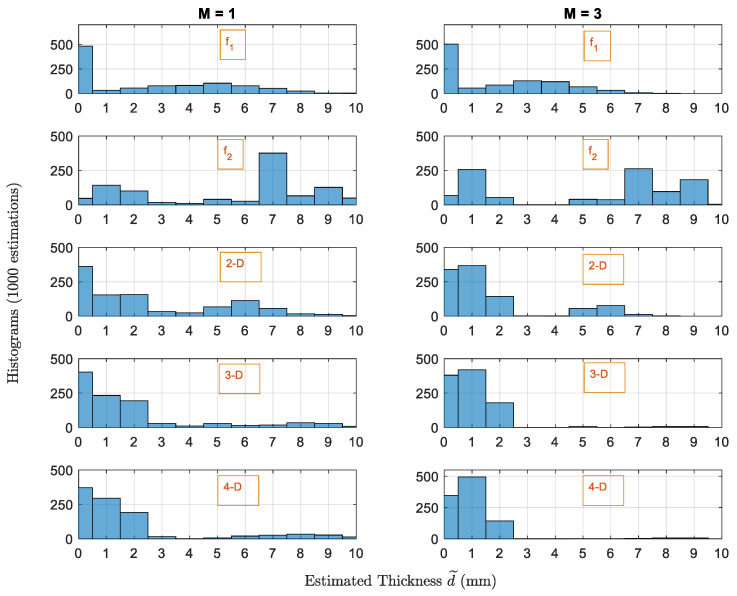
Histograms of the thicknesses estimated by 1D, 2D, 3D and 4D-estimators with single and multiple scans M=3. The actual thickness $d_{q}$ is 1 mm.

**Figure A5:**
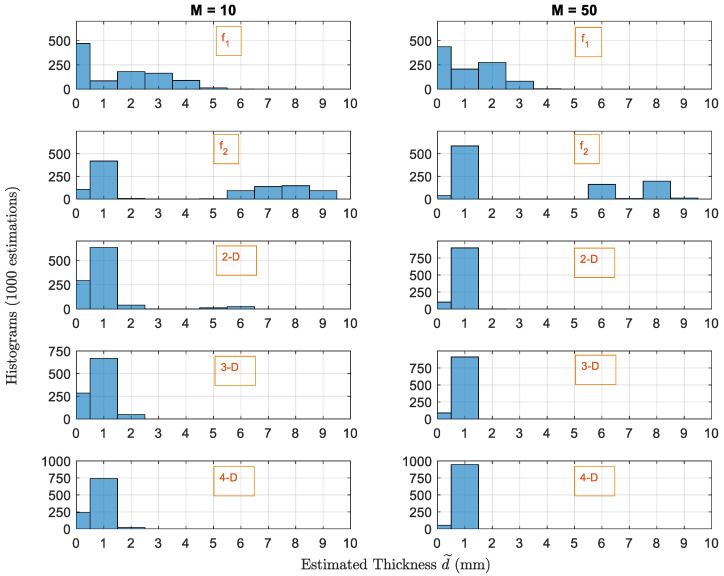
Histograms of the thicknesses estimated by 1D, 2D, 3D and 4D-estimators with multiple scans M=20, and 50. The actual thickness $d_{q}$ is 1 mm.

## Appendix A

Figure A1 shows the histograms of the thicknesses estimated by different estimators when the actual thickness is 10 mm. The single-frequency (1D) estimator uses $f_{1}=4$ GHz, and $f_{2}=12$ GHz. The dual-frequency (2D) estimator uses both $f_{1}$ and $f_{2}$ which are the optimal frequency pair for 10 mm as indicated in Table 1. The 3D estimator uses an additional frequency $f_{3}=7$ GHz. The 4D estimator uses $f_{1},f_{2},f_{3}$ in addition to $f_{4}=10$ GHz. At single scan (*M* = 1), the 1D estimator provides good results ($P_{c}=64\%$) due to the monotonic behavior of reflectivity at $f_{1}$. However, the cyclic behavior of reflectivity at $f_{2}$ decreases $P_{c}$ to 22% and increases $\varrho_{max}$ to 10 mm. The 2D, 3D, and 4D estimators increase $P_{c}$ to 65%,68%, and 70%, respectively. However, they fail to remove the error where $\varrho_{max}=8$ mm even though its probability is very low. When the number of scans increases to 3, the performance of all estimators is improved. $P_{e}$ decreases to 11% using the 4D estimator with $\varrho_{max}=1$ mm. Figure A2 provides a map of estimated thicknesses using colors, of a scanned 10 × 10 grid where 100 estimations are performed for 100 adjacent locations.

Similar performance is obtained for other thickness values. Table A1 shows the statistics (probability of error $P_{e}$ as %) obtained by the different estimators for multiple actual thicknesses. For each thickness, $P_{e}$ decreases when the number of used frequencies in the estimation process is increased. Moreover, doing more scans with the same frequencies will reduce the effect of the noise which yield to better performance for the estimators.

**Table A1:** Percentage error obtained by the all estimators under different scenarios.

$P_{e}\left(\%\right)$	*M*	$f_{1}$	$f_{2}$	2-D	3-D	4-D
d = 10 mm	1	46	78	35	32	30
	3	42	63	14	13	11
d = 5 mm	1	85	94	83	65	47
	3	77	91	58	33	20
d = 1 mm	1	97	85	83	77	72
	3	94	75	65	56	49
	10	90	58	36	32	26
	50	79	41	10	9	6

For instance, referring to Figure A3 when the tested thickness is 5 mm, the 2D estimator with 3 scans still provides thickness values from all possible values of $d_{range}$. The 4D estimator with 3 scans decreases $\varrho_{max}$ to 4 mm with $P_{c}=80\%$. Figure A4 and Figure A5 show the histograms of the thicknesses estimated when the actual thickness *d* is 1 mm for multiples number of scans (M=1,3,10, and 50). The 3D estimator with 3 scans still provides high probability for 0 mm oil (which means that no oil exists). The 4D estimator with 50 scans decreases $\varrho_{max}$ to 1 mm with $P_{c}=94\%$.
